# Vitamin D Inhibits Human Immunodeficiency Virus Type 1 and *Mycobacterium tuberculosis* Infection in Macrophages through the Induction of Autophagy

**DOI:** 10.1371/journal.ppat.1002689

**Published:** 2012-05-10

**Authors:** Grant R. Campbell, Stephen A. Spector

**Affiliations:** 1 Department of Pediatrics, Division of Infectious Diseases, University of California San Diego, La Jolla, California, United States of America; 2 Rady Children's Hospital, San Diego, California, United States of America; University of New Mexico, United States of America

## Abstract

Low vitamin D levels in human immunodeficiency virus type-1 (HIV) infected persons are associated with more rapid disease progression and increased risk for *Mycobacterium tuberculosis* infection. We have previously shown that 1*α*,25-dihydroxycholecalciferol (1,25D3), the active form of vitamin D, inhibits HIV replication in human macrophages through the induction of autophagy. In this study, we report that physiological concentrations of 1,25D3 induce the production of the human cathelicidin microbial peptide (CAMP) and autophagic flux in HIV and *M. tuberculosis* co-infected human macrophages which inhibits mycobacterial growth and the replication of HIV. Using RNA interference for *Beclin-1* and the *autophagy*-*related 5 homologue*, combined with the chemical inhibitors of autophagic flux, bafilomycin A_1_, an inhibitor of autophagosome-lysosome fusion and subsequent acidification, and SID 26681509 an inhibitor of the lysosome hydrolase cathepsin L, we show that the 1,25D3-mediated inhibition of HIV replication and mycobacterial growth during single infection or dual infection is dependent not only upon the induction of autophagy, but also through phagosomal maturation. Moreover, through the use of RNA interference for CAMP, we demonstrate that cathelicidin is essential for the 1,25D3 induced autophagic flux and inhibition of HIV replication and mycobacterial growth. The present findings provide a biological explanation for the benefits and importance of vitamin D sufficiency in HIV and *M. tuberculosis*-infected persons, and provide new insights into novel approaches to prevent and treat HIV infection and related opportunistic infections.

## Author Summary

Macroautophagy (autophagy - ‘*self-eating*’, lysosome-dependent degradation and recycling of the intracellular components in response to stress) is an important host defense mechanism against viral and mycobacterial infections. Recent studies have described that activation of autophagy in macrophages reduces the viability of *Mycobacterium tuberculosis* and HIV due to an intimate autophagy-phagocytosis interaction. Low serum levels of the 25-hydroxycholecalciferol form of vitamin D have been associated with an increased risk for active tuberculosis and HIV disease progression as well as *M. tuberculosis* susceptibility. In this study, we report that the active form of vitamin D, 1*α*,25-dihydroxycholecalciferol inhibits the replication of HIV and *M. tuberculosis* in a concentration dependent manner. Moreover, by inhibiting key stages in the autophagy pathway, we demonstrate that the inhibition of HIV and mycobacterial growth during single infection or dual infection is dependent not only upon the induction of autophagy, but also through phagosomal maturation. Furthermore, through the use of RNA interference for the human cathelicidin microbial peptide we demonstrate that cathelicidin is essential for the 1*α*,25-dihydroxycholecalciferol induced autophagic flux and inhibition of HIV replication and mycobacterial growth. These findings suggest that the induction of autophagy has the potential to be useful in the treatment of persons co-infected with HIV and *M. tuberculosis*.

## Introduction

Human immunodeficiency virus type-1 (HIV) is a global health problem that has infected 60 million people and caused 25 million deaths worldwide. Currently, there are an estimated 33 million people living with HIV including 2 million children. Despite the immune defense mechanisms that the host deploys against HIV and improved antiretroviral therapies, the virus persists in long lived cells including resting T cells, macrophages and dendritic cells. One-third of HIV-infected individuals are co-infected with *Mycobacterium tuberculosis*, a leading cause of death among people living with HIV. It has been proposed that the increase in *M. tuberculosis* pathology associated with HIV infection is caused by the disruption of the local immune response within the tuberculosis granulomas, decreasing their ability to contain *M. tuberculosis* leading to increased mycobacterial replication, dissemination and clinical disease [1]–[5]. Several studies have linked vitamin D deficiency (25-hydroxycholecalciferol (25D3) deficiency) with an increased risk for susceptibility to tuberculosis and active disease both in the presence [6] and absence of HIV infection [7]–[14]. Few studies have examined the association between vitamin D status and HIV disease progression and survival. However, the data available suggest that HIV-infected individuals have lower levels of 25D3 and/or the vitamin D3 active metabolite, 1*α*,25-dihydroxycholecalciferol (1,25D3) than uninfected individuals [15]–[21] with the lowest concentrations found in persons with AIDS [19], [20]. Additionally, women with low levels of 25D3 have an increased risk of HIV disease progression [22] and infants born to HIV-infected mothers with low 25D3 levels have an increased risk of HIV infection and increased mortality [23]. Although 25D3 has no direct anti-mycobacterial or antiretroviral effect, its hormonally active form, 1,25D3, modulates the immune response and has been shown to exert both anti-mycobacterial [24]–[27] and anti-HIV effects [27], [28] *in vitro*.

Macroautophagy (herein referred to as autophagy) is a trafficking pathway whereby cytoplasmic constituents such as sub-cellular organelles and microbial pathogens are engulfed by autophagosomes which fuse with lysosomes, forming autolysosomes, degrading the engulfed components. As an obligatory intracellular parasite, HIV survival is dependent upon its ability to exploit host cell machinery for replication and dissemination and to circumvent cellular processes that prevent its growth. During infection, HIV down regulates Beclin-1 and microtubule-associated protein 1 light chain 3B (LC3B)-II, reducing both basal autophagy and the numbers of autophagosomes per cell [29], [30]. However, silencing of autophagy proteins inhibits HIV infection of HeLa cells [31] and macrophages [28]. *M. tuberculosis* interferes with the biogenesis of phagolysosomes, and persists and replicates in macrophages within special immature phagosomes characterized by the exclusion of the vacuolar H^+^ ATPase and the absence of lysosomal hydrolases. In this study, we investigated the effect of 1,25D3 on productive HIV and *M. tuberculosis* infection of macrophages. We demonstrate that 1,25D3 inhibits HIV replication and mycobacterial growth using autophagic machinery.

## Results

### 1,25D3 Inhibits HIV replication in human macrophages in the presence of *M. tuberculosis* infection

**Figure 1:**
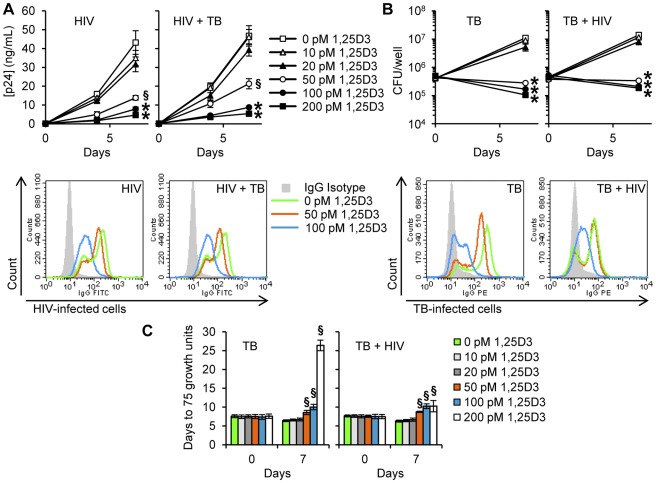
1,25D3 inhibits HIV and *M. tuberculosis* replication. MDM were incubated with 1,25D3 for 4 h before infection with HIV and/or *M. tuberculosis* (TB) for 3 h, washed then incubated with or without 1,25D3 for 7 days. (A) Top, extracellular release of HIV p24 antigen into the cell supernatant at days 0, 4 and 7 was detected by ELISA. Bottom, MDM were harvested and stained for HIV p17. *Histograms* are shown for a representative donor. (B) Top, cells were lysed after 3 h exposure to infectious agents or at the completion of the infection phase. Intracellular mycobacteria were harvested and assayed for mycobacterial growth at day 0 and day 7. Bottom, MDM were harvested and stained for mycobacterium. *Histograms* are shown for a representative donor. (C) Cell lysates from panel B were diluted and run in a MGIT 960. Bar and line graphs are reported as mean ± s.e.m. of three independent experiments performed in triplicate. § *P*<0.05; * *P*<0.001.

Previous studies have demonstrated that physiological concentrations of 1,25D3 have indirect antimicrobial activity against *M. tuberculosis* and HIV. However, to date, no study has assessed the ability of physiological levels of 1,25D3 to inhibit HIV during *M. tuberculosis* co-infection. Therefore, we initially assessed whether 1,25D3 inhibits HIV replication in macrophages by comparing the extent to which 1,25D3 pre-treatment affects HIV p24 antigen accumulation in the supernatants of macrophages that were subsequently infected with HIV and/or *M. tuberculosis*. 1,25D3 induced a dose-dependent inhibition of HIV replication with 50 pmol/L being the minimum concentration required to significantly inhibit HIV by day 7 (68% reduction; *P* = 0.003; Figure 1A), while 100 pmol/L, the concentration found in healthy 25D3-sufficient HIV-uninfected plasma, inhibited HIV by 82% (*P*<0.001; Figure 1A). Moreover, 1,25D3 induced a dose-dependent reduction in cells expressing HIV p17 (Figure 1A). In the presence of *M. tuberculosis* co-infection, 1,25D3 also induced a dose-dependent inhibition of HIV replication. The minimum dose of 1,25D3 required to significantly inhibit HIV replication did not change (54% reduction; *P* = 0.005; Figure 1A) and the profile of intracellular HIV p17 expression post-1,25D3 treatment was similar to *M. tuberculosis*-unexposed cells (Figure 1A).

### 1,25D3 Inhibits M. tuberculosis in human macrophages in the presence of HIV infection

To evaluate the capacity of 1,25D3 to suppress the mycobacterial growth in HIV-infected macrophages, MDM were infected with *M. tuberculosis* H37Rv at infection ratios (*M. tuberculosis*∶target cell) of 8∶1. Cells were then cultured for 7 d in the presence of varying concentrations of 1,25D3 after which they were lysed and the number of colony forming units (cfu) of *M. tuberculosis* in macrophages enumerated. There were no statistically differences observed in cfu at day 0. However, at day 7, 1,25D3 induced a dose-dependent reduction in cfu counts that became significant at 50 pmol/L (*P*<0.001; Figure 1B). This observed effect was dependent upon macrophage infection by *M. tuberculosis* as 1,25D3 had no effect on mycobacterial viability when grown in cell culture media or Middlebrook 7H9 broth alone (data not shown). Moreover, 1,25D3 also induced a dose-dependent reduction in *M. tuberculosis*-positive cells as assessed by flow cytometry (Figure 1B). The effect of HIV infection on the capacity of macrophages to limit mycobacterial growth was next assessed. Although the presence of HIV resulted in a slightly increased cfu count at day 7, there were no significant differences both in cfu count or in growth index (cfu day 7∶cfu day 0) compared with *M. tuberculosis* only treated cells. In the presence of HIV infection, 1,25D3 treatment was associated with a dose-dependent reduction in mycobacterial viability that became statistically significant at 50 pmol/L, at which point the cfu count was less than at day 0 (8.9×10^4^ versus 7.5×10^4^ cfu/well; *P* = 0.001; Figure 1B). Flow cytometry also revealed that 100 pmol/L 1,25D3 significantly inhibited mycobacterial growth (Figure 1B). Dilutions of the lysates were also run in a Mycobacteria Growth Indicator Tube 960 apparatus (MGIT 960). 1,25D3 induced a dose-dependent increase in time to positive (75 growth units) both in the presence and in the absence of HIV infection indicating a smaller initial mycobacterial count post 1,25D3 treatments (Figure 1C).

### 1,25D3 Induces autophagy in human macrophages in the presence of *M. tuberculosis* and HIV-co-infection

**Figure 2:**
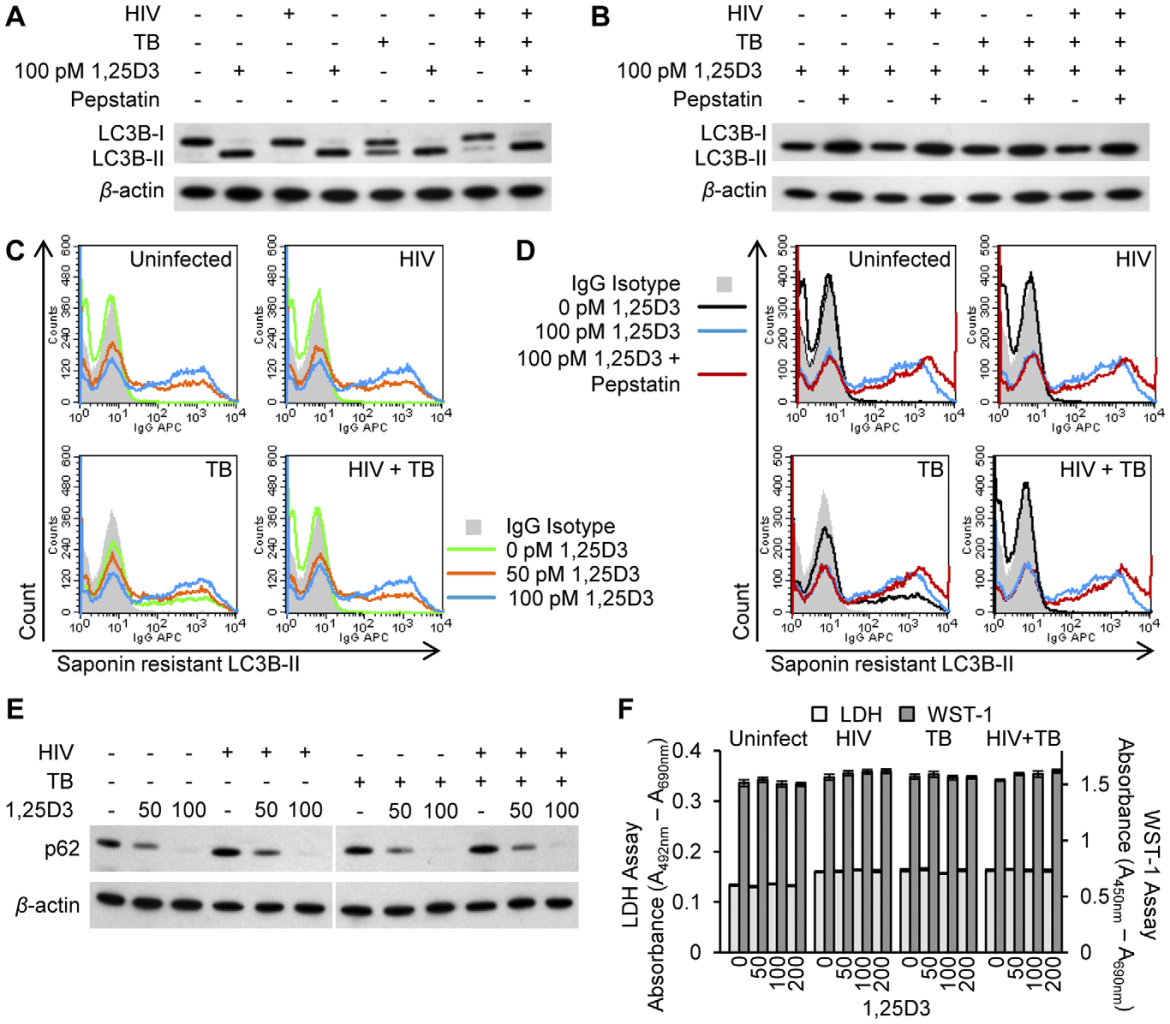
1,25D3 induces autophagy in human macrophages co-infected with HIV and *M. tuberculosis*. HIV and/or *M. tuberculosis* (TB) infected MDM were treated for 7 days with 100 pmol/L 1,25D3. (A) Cells were lysed and immunoblots of LC3B isoforms using antibody to LC3B or *β*-actin performed. (B) Cells were incubated with 10 µg/mL pepstatin A for 4 h on day 7 prior to lysis. Immunoblots of LC3B isoforms using antibody to LC3B or *β*-actin. (C) Flow cytometry analysis of saponin-resistant LC3B-II in macrophages after 1,25D3 treatment for 7 d. Representative histograms of cells displaying saponin-resistant LC3B-II from three donors are shown. (D) Flow cytometry analysis of saponin-resistant LC3B-II in macrophages after 1,25D3 treatment for 7 d followed by 10 µg/mL pepstatin A for a further 4 h. Representative histograms of cells displaying saponin-resistant LC3B-II from three donors are shown. (E) Immunoblots of p62 using antibody to p62 or *β*-actin 7 d after macrophages treated with 1,25D3. (F) At 7 d post-infection, aliquots of supernatant taken before the addition of WST-1 were tested for lactate dehydrogenase (LDH) spectrophotometrically using the LDH^PLUS^ assay. For the last hour cells were incubated with WST-1, and the reduction of the WST-1 reagent to its formazan product was monitored spectrophotometrically.

It has previously been demonstrated that 1,25D3 induces autophagy in human macrophages during infection with either HIV or *M. tuberculosis*. However, no study has investigated the effect of 1,25D3 on autophagy during co-infection. Therefore, the ability of 1,25D3 to induce autophagy in HIV and *M. tuberculosis* co-infected human macrophages was assessed. An established molecular marker for the induction of autophagy is the degree of LC3B lipidation [32]. During autophagy, cytosolic LC3B-I is converted to LC3B-II by a ubiquitin-like system that involves autophagy related protein-7 (ATG7), ATG3 and the ATG5-ATG12 complex. The ATG5-ATG12 complex ligates LC3B-II to the nascent autophagosome membrane through phosphatidylethanolamine with the LC3B-II associated with the inner membrane degraded after fusion of the autophagosome with lysosomes. Therefore, the conversion of LC3B-I to LC3B-II and its turnover is an indicator of autophagy induction and flux [32]. 1,25D3 treatment of MDM induced an increase in LC3B-II in cells that were incubated with HIV and/or *M. tuberculosis* (Figure 2A). The accumulation of LC3B-II was increased in the presence of the lysosomal protease inhibitor pepstatin A regardless of infection status (Figure 2B), indicative of autophagic flux [33].

When autophagosomes are formed, LC3B redistributes from a soluble diffuse cytosolic pattern to an insoluble autophagosome-associated vacuolar pattern [34], [35] allowing the quantification of autophagosome-associated LC3B-II in human macrophages using saponin resistance and flow cytometry [35]. Staining for endogenous LC3B in saponin washed macrophages revealed that the percentage of cells containing a saponin resistant fraction significantly increased with dose of 1,25D3 (*P*<0.05; Figure 2C). In uninfected cells and in HIV and/or *M. tuberculosis* infected cells, co-treatment with pepstatin A significantly increased the percentage and fluorescent signal of cells containing a saponin resistant fraction upon treatment with 100 pmol/L 1,25D3 indicative of autophagic flux (*P*<0.05; Figure 2D). Another control to confirm that the increase in LC3B-II observed in 1,25D3 treated cells during HIV and/or *M. tuberculosis* infection represents increased autophagic flux, rather than an accumulation of LC3-positive autophagosomes is the measurement of polyubiquitin-binding protein p62 (sequestosome 1) degradation through immunoblotting as p62 binds LC3B [33]. Inhibition of autophagy leads to an increase in p62 protein levels while p62- and LC3-positive bodies are degraded in autolysosomes during autophagic flux [36]. HIV and/or *M. tuberculosis* infected MDM were subjected to 1,25D3 stimulus for 7 d after which p62 protein levels were measured by western blot analysis. 1,25D3 treatment induced a decrease in p62 protein levels in HIV and/or *M. tuberculosis* infected MDM compared to vehicle-treated cells, corresponding to a stimulation of autophagic flux (Figure 2E).

Although there was an increase in autophagic markers in the absence of visible pyknosis, karyorrhexis, or plasma membrane blebbing, it was important to confirm that the cells were not undergoing cell death at the physiological concentrations being used, as the induction of excessive autophagy can cause cell death in mammalian cells in experimental systems *in vitro* [37]. Therefore, plasma membrane breakdown (as a sign of cytotoxicity) using the lactate dehydrogenase assay was measured in combination with the WST-1 assay that measures the activity of the mitochondrial respiratory chain (as an indicator of viable cells). After 7 d at physiological concentrations, 1,25D3 exhibited no cytotoxic effects in the presence or absence of HIV and/or *M. tuberculosis* infection (*P*>0.05; Figure 2F).

### 1,25D3-Mediated autophagy inhibits the replication of HIV and M. tuberculosis in co-infected macrophages

**Figure 3:**
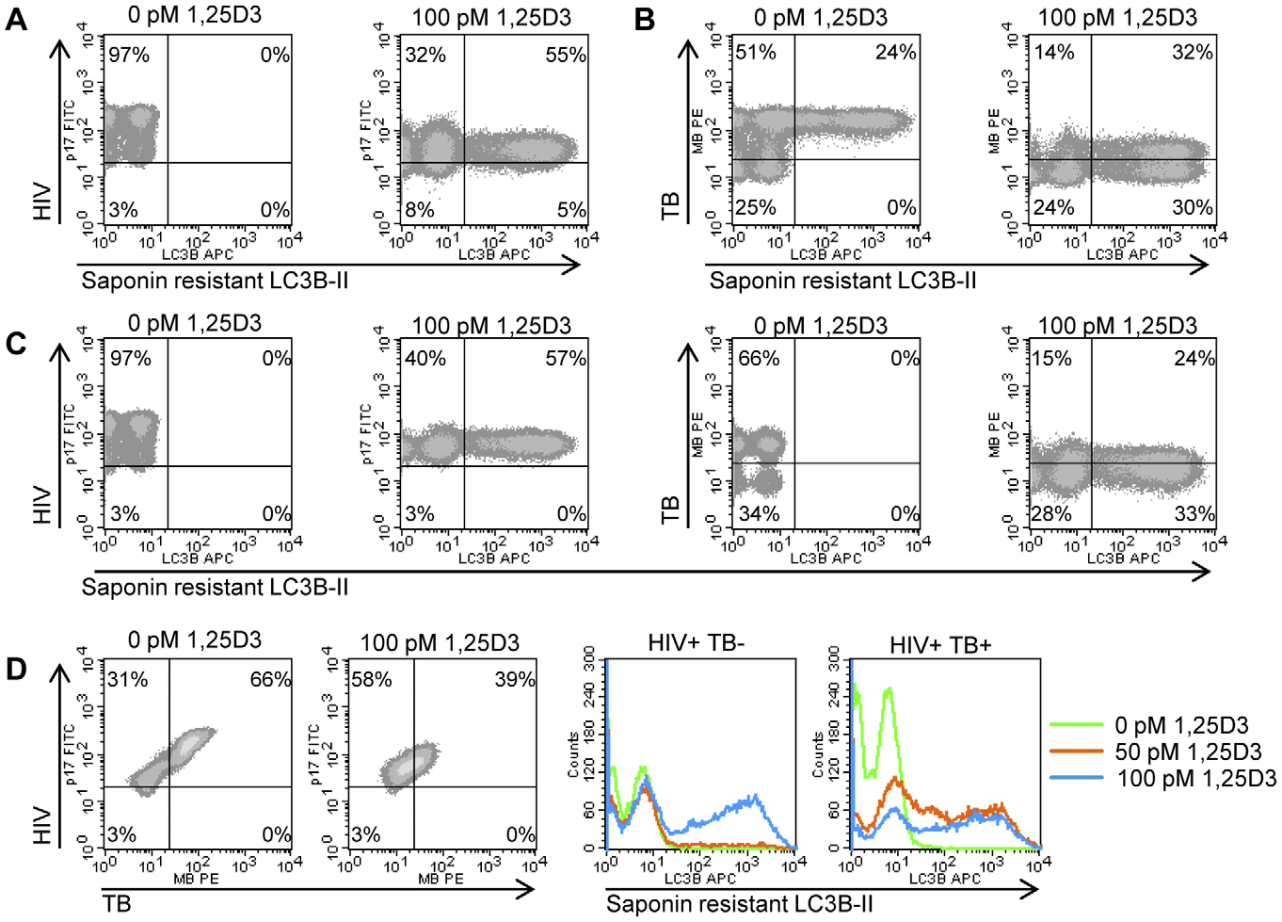
1,25D3 induces autophagy in human macrophages co-infected with HIV and *M. tuberculosis*. HIV (A), *M. tuberculosis* (TB) (B), and HIV/TB dual infected MDM (C) were treated for 7 days with 100 pmol/L 1,25D3, harvested and stained for HIV p17, mycobacteria, and saponin-resistant LC3B-II. Representative density plots from three donors are shown. (D) HIV/*M. tuberculosis* infected MDM (C) were treated for 7 days with 100 pmol/L 1,25D3, harvested and stained for HIV p17, mycobacteria, and saponin-resistant LC3B-II. Left, representative density plots from three donors from HIV/*M. tuberculosis* infected MDM are shown for HIV/*M. tuberculosis* co-infection. Right, histograms of saponin-resistant LC3B-II in macrophages that were HIV^+^ *M. tuberculosis* ^−^ or HIV^+^ *M. tuberculosis* ^+^ at day 7 post-infection.

Recent studies have demonstrated that physiological levels of 1,25D3 inhibit *M. tuberculosis* [38] and HIV [28] through autophagy dependent mechanisms. Therefore, it was important to investigate the relationship between infection status and the 1,25D3-mediated induction of autophagy. At 7 d post- HIV infection, the majority of MDM were positive for HIV p17 (Figure 3A). Treatment with 100 pmol/L 1,25D3, reduced the number of cells with detectable HIV p17 and was accompanied by an overall reduction in mean HIV p17 antibody fluorescence that was greatest in cells that also exhibited saponin resistant LC3B-II (Figure 3A). In the absence of 1,25D3 and HIV infection, incubation of MDM with *M. tuberculosis* for 7 days resulted in 75% of MDM becoming positive for *M. tuberculosis*, one third of which were also positive for saponin resistant LC3B-II (Figure 3B). All *M. tuberculosis* negative cells were saponin resistant LC3B-II negative. 100 pmol/L 1,25D3 significantly decreased the number of *M. tuberculosis* positive cells (*P* = 0.002; Figure 3B) while concomitantly increasing the number of saponin resistant LC3B-II positive cells. Co-culture of MDM with both HIV and *M. tuberculosis* for 7 days resulted in the inhibition of saponin resistant LC3B-II that appeared in *M. tuberculosis*-only infected MDM. Similar to HIV and *M. tuberculosis*-only infections, incubation with 100 pmol/L 1,25D3 increased the numbers of saponin resistant LC3B-II positive cells while reducing the number of *M. tuberculosis* positive but not HIV p17 positive cells (Figure 3C and 3D). Cells co-infected with HIV/*M. tuberculosis* demonstrated saponin resistant LC3B-II when treated with 50 pmol/L 1,25 D3, while cells infected with HIV did not show saponin resistant LC3B-II when treated with 50 pmol/L 1,25 D3. (Figure 3D).

**Figure 4:**
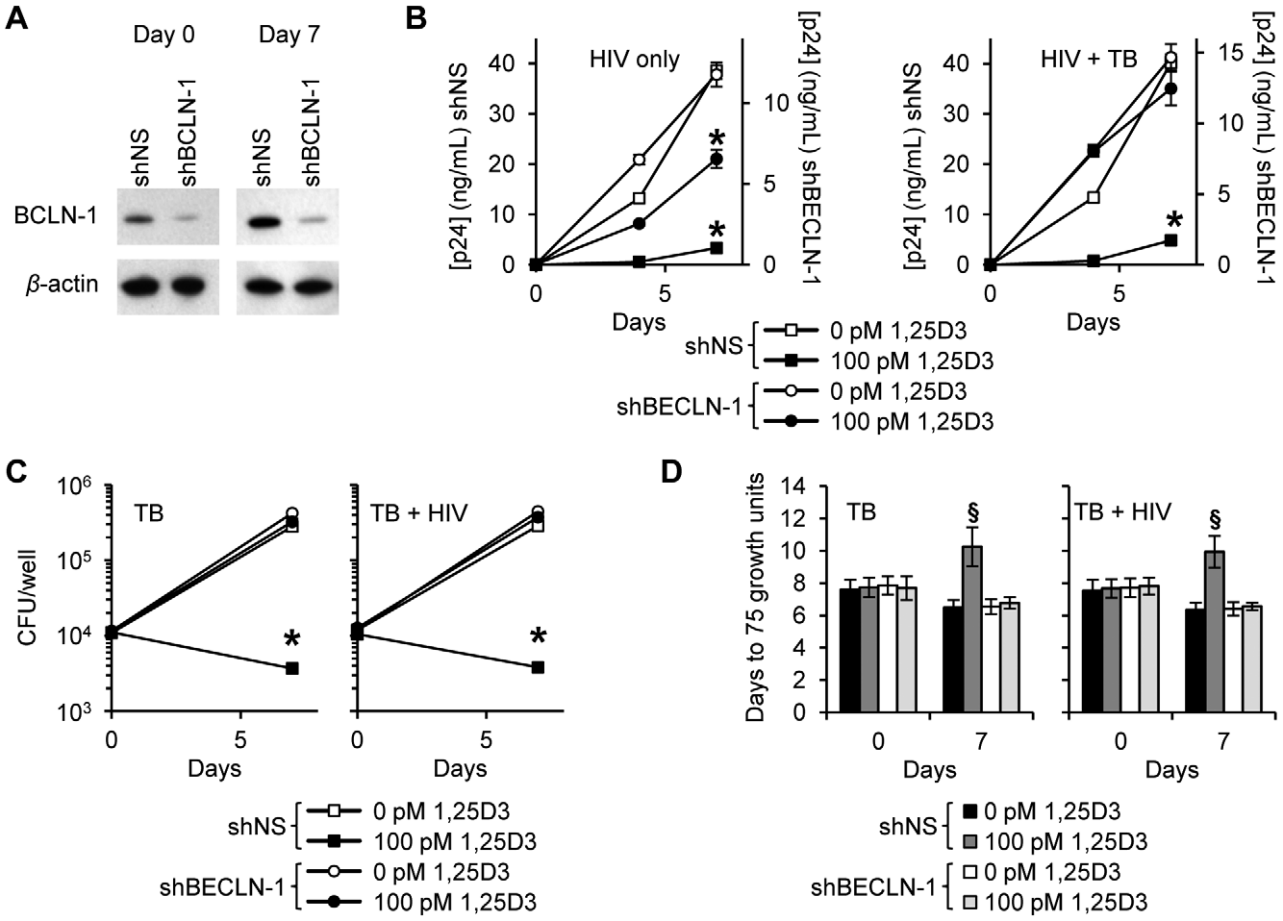
1,25D3 inhibition of HIV and *M. tuberculosis* is Beclin-1 dependent. MDM were transduced with non-specific scrambled shRNA (shNS) or Beclin-1 shRNA (shBCLN1) and selected using puromycin resistance. Five days later, cells were incubated with 100 pmol/L 1,25D3 or vehicle control for 4 h before infection with HIV and/or *M. tuberculosis* (TB) for 3 h. Cells were then washed and incubated with 100 pmol/L 1,25D3 or vehicle control for 7 days. (A) Immunoblot analysis performed using antibodies raised to Beclin-1 or *β*-actin after initial pathogen exposure (Day 0) or after 7 days. (B) ELISA performed for HIV p24 antigen release over 7 d. (C) Intracellular mycobacteria were harvested and assayed for mycobacterial growth by cfu enumeration at day 0 and day 7. (D) Intracellular mycobacteria harvested and assayed for viability based on time to positivity (75 growth units) at day 0 and day 7 using the MGIT 960. All bar and line graphs are reported as mean ± s.e.m. of three independent experiments performed in triplicate. § *P*<0.05; * *P*<0.001.

The contribution of 1,25D3-induced autophagy in 1,25D3-mediated inhibition of HIV and mycobacterial growth was investigated by inhibiting sequential steps of the autophagy pathway. As the physical interaction of class III phosphatidylinositol 3-kinase with Beclin-1 forms the phosphatidylinositol 3-kinase class III kinase complex and this complex is essential for the induction of autophagosome formation at the vesicle elongation step, RNAi for Beclin-1 was initially employed. Concomitant with the findings that HIV utilizes autophagic machinery for replication [28], [31], [39], the supernatant p24 antigen concentration in Beclin-1 silenced cells was decreased in the absence of 1,25D3 regardless of *M. tuberculosis* infection status. In the absence of *M. tuberculosis* infection, Beclin-1 silencing (Figure 4A) significantly reversed the 1,25D3 mediated inhibition of HIV at day 7 from 91% to 44% (*P*<0.001; Figure 4B). Conversely, in the presence of *M. tuberculosis* infection, Beclin-1 silencing reduced 1,25D3 inhibition of HIV infection from 88% to 15% (*P*<0.001; Figure 4B).

In the absence of HIV, Beclin-1 silencing increased mycobacterial growth 51% at day 7 increase compared with the scrambled non-target control RNAi treated cells (*P* = 0.0006). HIV co-infection increased this difference to 55% (*P*<0.001) although the difference between the cfu counts between the *M. tuberculosis* and HIV/*M. tuberculosis* treated Beclin-1 RNAi transduced cells was not significant (*P* = 0.36). The effect of 1,25D3 on the capacity of macrophages to limit mycobacterial growth was next assessed. Beclin-1 silencing protected against the anti-mycobacterial effects of 1,25D3 both in the presence and in the absence of HIV infection (*P*>0.05; Figure 4C). A similar profile was observed using the MGIT 960 (Figure 4D).

**Figure 5:**
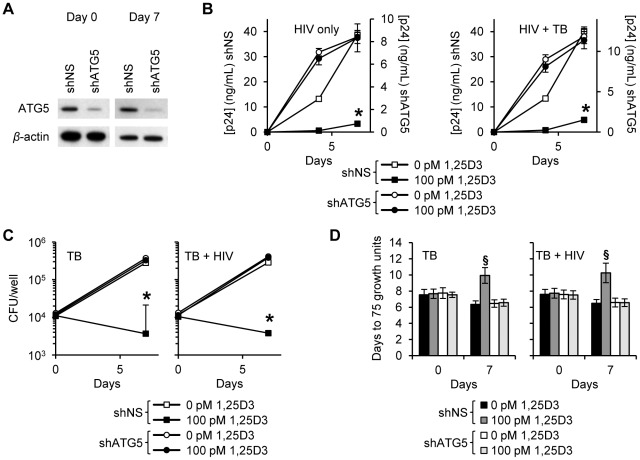
1,25D3 inhibition of HIV and *M. tuberculosis* is ATG5 dependent. MDM were transduced with non-specific scrambled shRNA (shNS) or ATG5 shRNA (shATG5) and selected using puromycin resistance. Five days later, cells were incubated with 100 pmol/L 1,25D3 or vehicle control for 4 h before infection with HIV and/or *M. tuberculosis* (TB) for 3 h. Cells were then washed and incubated with 100 pmol/L 1,25D3 or vehicle control for 7 days. (A) Immunoblot analysis performed using antibodies raised to ATG5 or *β*-actin after initial pathogen exposure (Day 0) or after 7 days. (B) ELISA performed for HIV p24 antigen release over 7 d. (C) Intracellular mycobacteria were harvested and assayed for mycobacterial growth by cfu enumeration at day 0 and day 7. (D) Intracellular mycobacteria harvested and assayed for viability based on time to positivity (75 growth units) at day 0 and day 7 using the MGIT 960. All bar and line graphs are reported as mean ± s.e.m. of three independent experiments performed in triplicate. § *P*<0.05; * *P*<0.001.

During autophagy, cytosolic LC3B-I is converted to LC3B-II by an ubiquitin-like system that involves the autophagy-related 7 (ATG7) homologue, ATG3 and the ATG5-ATG12 complex. The ATG5-ATG12 complex ligates LC3B-II to the nascent autophagosome membrane through phosphatidylethanolamine. Therefore, RNAi of ATG5 inhibits autophagosome formation. In agreement with the above findings that HIV was inhibited through Beclin-1 silencing, ATG5 silencing (Figure 5A) also had an inhibitory effect on HIV replication alone and in the presence of *M. tuberculosis* co-infection (Figure 5B). In the absence of *M. tuberculosis* co-infection, ATG5 RNAi completely abrogated the 1,25D3 mediated inhibition of HIV by day 7 (*P*<0.001; Figure 5B). In the presence of *M. tuberculosis* co-infection, ATG5 silencing reduced the 1,25D3 mediated inhibition of HIV at day 7 from 91% to 4% (*P*<0.001; Figure 5B). As for Beclin-1 RNAi, *M. tuberculosis* growth was significantly increased in HIV infected and uninfected ATG5 silenced cells in the absence of 1,25D3 treatment (*P*<0.003; Figure 5C). Moreover, the effect of 1,25D3 on the capacity of macrophages to limit mycobacterial growth was severely diminished both in the presence and absence of HIV infection (*P*<0.001; Figure 5C). A similar profile was observed using the MGIT 960. ATG5 RNAi abrogated the 1,25D3 inhibition of mycobacterial growth both in the absence and presence of HIV infection (Figure 5D).

**Figure 6:**
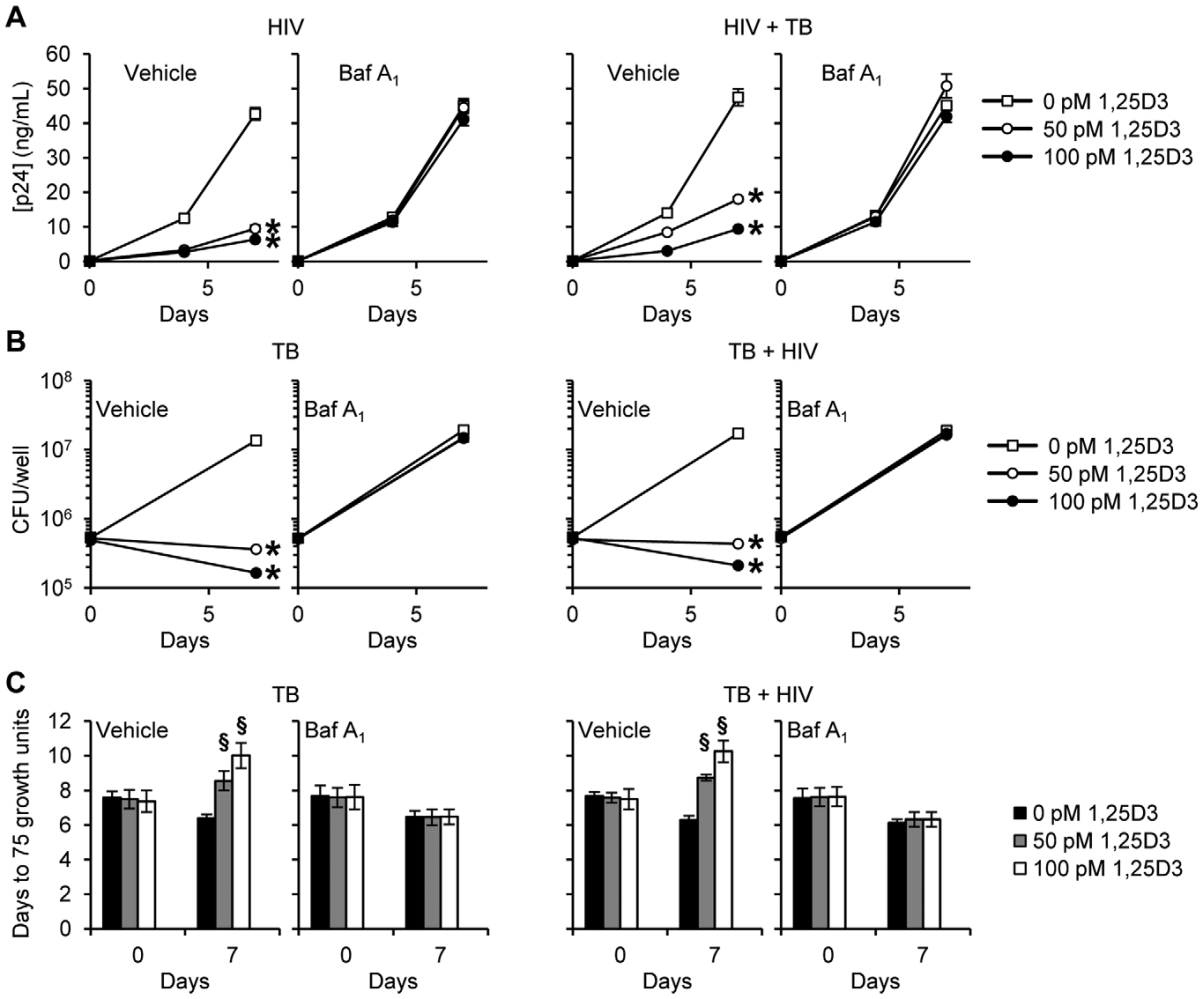
Bafilomycin A_1_ inhibits the 1,25D3 mediated inhibition of HIV and *M. tuberculosis* replication. MDM were pretreated with bafilomycin A_1_ (Baf A_1_) before treatment with 1,25D3 and subsequent infection with HIV and/or *M. tuberculosis* (TB). (A) ELISA performed for HIV p24 antigen release at days 0, 4 and 7. (B) Intracellular mycobacteria were harvested and assayed for mycobacterial growth by cfu enumeration at day 0 and 7. (C) Intracellular mycobacteria harvested and assayed for viability using the MGIT 960 based on time to positivity at day 0 and 7. All bar and line graphs are reported as mean ± s.e.m. of three independent experiments performed in triplicate. § *P*<0.05; * *P*<0.01.

We next investigated whether autophagosome acidification, a late stage event during autophagy, is required for the 1,25D3-mediated autophagic inhibition of HIV and *M. tuberculosis*. During autophagy, lysosomes fuse with autophagosomes to form autophagolysosomes. Macrophages were treated with bafilomycin A_1_, an inhibitor of the vacuolar H^+^ ATPase and thus autophagosome-lysosome fusion, and subsequently infected with HIV and/or *M. tuberculosis*. Bafilomycin A_1_ modestly increased HIV production over 7 d in the absence of 1,25D3 and *M. tuberculosis* infection although this was not significant. It had no effect on HIV replication in the presence of *M. tuberculosis* infection. In the presence of 1,25D3, bafilomycin A_1_ abrogated the 1,25D3-mediated inhibition of HIV both alone and in the presence of *M. tuberculosis* (Figure 6A). Mycobacterial growth was similarly increased after bafilomycin A_1_ treatment in the absence of 1,25D3 in both infection models although this was not significant. Bafilomycin A_1_ significantly reduced the inhibitory effect of 100 pmol/L 1,25D3 both in the presence (*P* = 0.05; Figure 6B) and absence of HIV co-infection (*P* = 0.06; Figure 6B). We obtained comparable results using the MGIT 960 with 100 pmol/L 1,25D3 significantly inhibiting mycobacterial growth both in the presence and absence of HIV infection (*P*<0.0001; Figure 6C). These results suggest that the acidic pH of autophagolysosomes is required for the autophagy-mediated control of both HIV replication and *M. tuberculosis* growth by 1,25D3.

**Figure 7:**
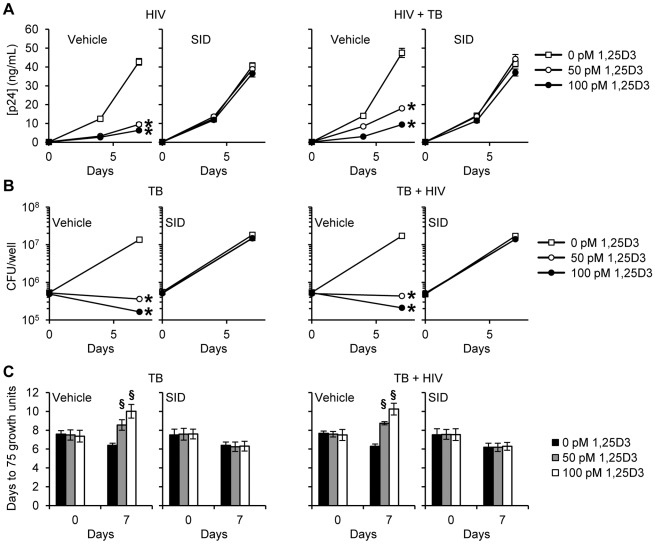
SID 26681509 inhibits the 1,25D3 mediated inhibition of HIV and *M. tuberculosis* replication. MDM were pretreated with SID 26681509 (SID) before treatment with 1,25D3 and subsequent infection with HIV and/or *M. tuberculosis* (TB). (A) ELISA performed for HIV p24 antigen release at days 0, 4 and 7. (B) Intracellular mycobacteria were harvested and assayed for mycobacterial growth by cfu enumeration at day 0 and 7. (C) Intracellular mycobacteria harvested and assayed for viability using the MGIT 960 based on time to positivity at day 0 and 7. All bar and line graphs are reported as mean ± s.e.m. of three independent experiments performed in triplicate. § *P*<0.05; * *P*<0.01.

After lysosomes fuse with autophagosomes to form autophagolysosomes, the sequestered components are then degraded by lysosomal hydrolases and presumably released into the cytosol by lysosomal efflux permeases. Therefore the effect of lysosomal hydrolases in 1,25D3-mediated inhibition of HIV and *M. tuberculosis* through autophagy was examined using SID 26681509, a novel thiocarbazate specific inhibitor of the lysosome hydrolase cathepsin L. In the absence of 1,25D3 there was no net inhibition of either HIV or *M. tuberculosis* growth (Figure 7A). Moreover, in the presence of 1,25D3, SID 26681509 abrogated the inhibition of both HIV and *M. tuberculosis* (Figure 7B and 7C). Together, these data indicate that the 1,25D3-mediated induction of autophagy in macrophages inhibits HIV and *M. tuberculosis* replication regardless of co-infection status.

### 1,25D3-Mediated autophagy and inhibition of HIV and *M. tuberculosis* is dependent upon the expression of human cathelicidin microbial peptide

**Figure 8:**
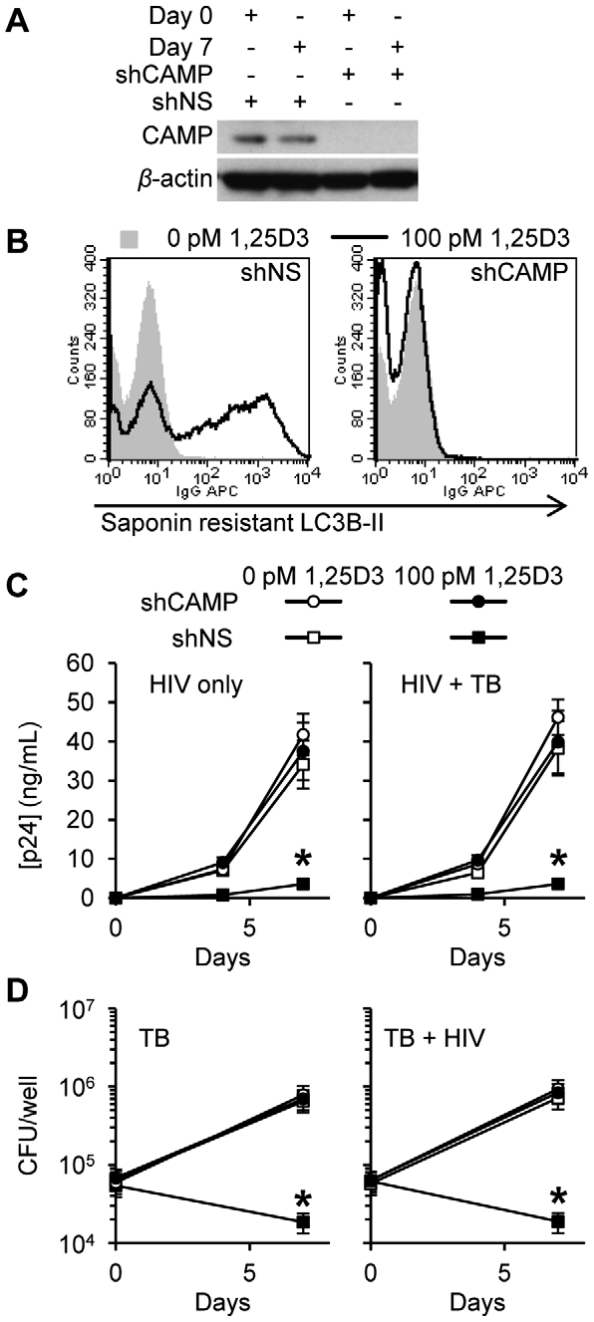
Inhibition of HIV and *M. tuberculosis* by 1,25D3 is CAMP and autophagy dependent. MDM were transduced with non-specific scrambled shRNA (shNS) or CAMP shRNA (shCAMP) and selected using puromycin resistance. Five days later, cells were incubated with 100 pmol/L 1,25D3 or vehicle control for 4 h before infection with HIV and/or *M. tuberculosis* (TB) for 3 h. Cells were then washed and incubated with 100 pmol/L 1,25D3 or vehicle control for 7 days. (A) Immunoblot analysis performed using antibodies raised to CAMP or *β*-actin after initial pathogen exposure (Day 0) or after 7 days. (B) Day 7 HIV-1_Ba-L_ and TB co-infected cells were harvested and stained for saponin-resistant LC3B-II at day 7. A representative histogram from three donors are shown. (C) ELISA performed for HIV p24 antigen release over 7 d. (D) Intracellular mycobacteria were harvested and assayed for mycobacterial growth by cfu enumeration at day 0 and day 7. All bar and line graphs are reported as mean ± s.e.m. of three independent experiments performed in triplicate. * *P*<0.001.

Previous studies have demonstrated that the human cathelicidin microbial peptide (CAMP) is required for the 1,25D3 mediated antimycobacterial activity against *M. tuberculosis* [38], [40] and the 1,25D3-mediated autophagy in human macrophages [38]. To address the role of CAMP in 1,25D3-induced antimicrobial activity, RNAi for CAMP was employed. Transduction of shCAMP into MDM completely silenced the 1,25D3-induced expression of CAMP during HIV and *M. tuberculosis* co-infection (Figure 8A). Moreover, CAMP silencing markedly inhibited 1,25D3-induced autophagy, whereas MDM transduced with a scrambled (shNS) showed increased saponin resistant LC3, consistent with autophagosome formation. Concomitant with our findings that autophagy is required for the restriction of HIV replication, CAMP silencing reduced 1,25D3 inhibition of HIV both alone (90% shNS versus 10% shCAMP; *P*<0.001; Figure 8C) and in the presence of *M. tuberculosis* infection (91% shNS versus 10% shCAMP; *P*<0.001; Figure 8C). Moreover, CAMP silencing completely blocked the 1,25D_3_-mediated antimycobacterial activity (*P*<0.001; Figure 8D). These data suggest that CAMP is required for 1,25D_3_-mediated antimicrobial activity against both intracellular *M. tuberculosis* and HIV in human MDM.

## Discussion

Despite much progress in the care and treatment of persons infected with HIV and those suffering from tuberculosis, the two pathogens are inextricably linked as important causes of morbidity and mortality worldwide. Thus, the effective treatment of persons co-infected with HIV and *M. tuberculosis* remains a major unmet challenge. In the case of tuberculosis, administration of vitamin D and/or sunlight exposure has a long and colorful history [11]. In fact, the beneficial effects of cod liver oil in the treatment of tuberculosis were first recognized in 1849 and Niels Finsen received the Nobel Prize in 1903 for his discovery of ultra-violet light as an effective therapy for the cutaneous form of tuberculosis, lupus vulgaris. However, more than 100 years later, the therapeutic benefits of vitamin D and the plasma levels of 25D3 required to provide clinical benefit remain controversial [41], [42].

Our results demonstrate that physiological concentrations of 1,25D3 inhibit both HIV and *M. tuberculosis* replication in human macrophages through an autophagy and CAMP dependent mechanism, and that 1,25D3 can act as a potent stimulator of innate antimicrobial responses. The ability of macrophages to kill intracellular pathogens is pivotal to the outcome of microbial infections. Within macrophages, *M. tuberculosis* and HIV reside in phagosomes and evade host microbial mechanisms by blocking phagosome maturation and fusion with lysosomes [39], [43]. However, the host can overcome this block through the induction of autophagy [28], [44], [45]. Previous studies have shown that physiological concentrations of 1,25D3 mediate the induction of autophagy and the individual killing of *M. tuberculosis* [38] and HIV [28]. To our knowledge the present study is the first to demonstrate that 1,25D3 inhibits both *M. tuberculosis* and HIV co-infection of macrophages through the induction of autophagy mediated by CAMP.

The *in vitro* findings reported here are supported by association studies that have linked low levels of 25D3 and/or 1,25D3 with increased risk of, or severity of infection with HIV [15]–[23] and *M. tuberculosis* [7]–[10], [12]–[14], [21]. Why HIV-infected individuals tend to have lower levels of 1,25D3 and/or 25D3 is largely unknown but is thought to be related to inadequate renal 1*α*-hydroxylation mediated by pro-inflammatory cytokines and/or a direct effect of antiretroviral drugs [16], [19], [46]. The effects of HIV viral products on 1,25D3 and/or 25D3 syntheses have not been evaluated. Vitamin D deficiency is conservatively defined by most experts as <50 nmol/L 25D3 [47]; 52–72 nmol/L 25D3 is considered to indicate insufficiency and >73 nmol/L considered sufficient [47]. In contrast to this, the estimated mean concentration of 25D3 present in people worldwide is just 54 nmol/L [48]. Four genes are known to contribute to the variability of serum 25D3 concentrations: 7-dehydrocholesterol reductase (involved in cholesterol synthesis and the availability of 7-dehydrocholesterol in the skin), 25-hydroxylase CYP2R1 (cytochrome P450, family 2, subfamily R, polypeptide 1) and CYP24A1 (cytochrome P450, family 24, subfamily A, polypeptide 1) (degrades and recycles 1,25D3) and GC (group-specific component [vitamin D binding protein]) which encodes for the vitamin D binding protein. Genetic variations at these loci were recently identified to be significantly associated with an increased risk of 25D3 insufficiency [49].

A better understanding of the pathogenesis of tuberculosis will be necessary if novel interventions are to be developed to effectively treat persons infected with *M. tuberculosis* and HIV. The induction of autophagy to enhance treatment is attractive for a number reasons including: 1) autophagy would work at the cellular level to improve intracellular killing of both pathogens; 2) enhanced autophagy is likely to be equally active against multi-drug resistant (MDR) and extensively drug resistant (XDR) *M. tuberculosis* as it is against drug sensitive *M. tuberculosis*; 3) autophagy has the potential to inhibit non-replicating HIV and *M. tuberculosis* within endosomes; 4) autophagy promotes innate and adaptive immunity; and 5) HIV or *M. tuberculosis* resistance is unlikely to develop.

The characterization of the 1,25D3 mediated antimicrobial mechanism in macrophages provides further evidence of the link between vitamin D and the immune system. The intracrine nature of this mechanism suggests that the ability of 25D3 to promote *M. tuberculosis* and HIV killing could be affected by the efficiency of the synthesis of 1,25D3 by macrophages. Unlike the parathyroid-hormone responsiveness of renal cytochrome P450, family 27, subfamily B, polypeptide 1 (CYP27B1), extra-renal CYP27B1 is not subject to the same feedback control so that the local synthesis of 1,25D3 in macrophages probably reflects the availability of 25D3. Toll-like receptor 2/1 agonists and interferon gamma upregulate the expression of the CYP27B1 which 1*α*-hydroxylates 25D3 into 1,25D3, activating and upregulating the expression of the vitamin D (1,25D3) receptor (VDR) leading to the induction of CAMP and autophagic flux [50], [51]. When serum is 25D3 deficient, TLR2/1 agonists (<25 nmol/L) [50] and interferon gamma (<45 nmol/L) [51] are unable to induce the expression of CAMP from monocytes or macrophage.

In the present study we demonstrate that the 1,25D3-induced autophagy and anti-mycobacterial activity was dependent on the expression of endogenous CAMP in agreement with previous findings [38]. Moreover, by using RNAi for CAMP we demonstrate, for the first time, that the 1,25D3 induced autophagy and subsequent autophagy-induced antiretroviral activity is dependent upon endogenous CAMP expression. This finding highlights the importance of endogenous CAMP during autophagy induction and antiretroviral activity. High concentrations of exogenous synthetic cleaved form of CAMP (LL-37) have been shown to reduce mycobacterial growth [25], [38], [40] and HIV replication [28]. However, at the much lower concentrations secreted *in vitro* by 1,25D3 treated macrophages, exogenously added LL-37 has not been shown to have an inhibitory effect on either organism [25], [28] or induce the formation of autophagosomes [28]. However, Yuk *et al.* [38] clearly showed that CAMP is upstream of Beclin-1 and ATG5 induction post-1,25D3 treatment and we show that in CAMP silenced cells 1,25D3 fails to induce LC3B lipidation. Further work is necessary to determine the precise role CAMP has in 1,25D3 induced autophagy and antimicrobial activity.

When modeling the replication of mycobacteria in human cells, we used the standard protocol of incubating macrophages with *M. tuberculosis* followed by washing the cells of non-phagocytosed bacilli. In this way, the intracellular replication of mycobacteria in macrophages can be assessed. However, *M. tuberculosis* is able to replicate extracellularly *in vivo* and as would be expected, we observed that 1,25D3 had no effect on *M. tuberculosis* survival in the extracellular compartment. In addition, as the cfu assay is cumbersome for high-throughput analyses, we adopted and ran samples in the MGIT 960 using a standard curve derived from the untreated macrophage cell lysates and found that the results obtained with this method were closely aligned with those obtained using the more subjective cfu method. This procedure can be used to more rapidly and efficiently screen compounds for the ability to inhibit *M. tuberculosis* replication compared to the standard assay.

In summary, this study establishes a role for autophagy during the early phases of HIV infection and demonstrates that the induction of autophagy by 1,25D3 can inhibit HIV and *M. tuberculosis* co-infection in macrophages. Well-controlled clinical trials are needed to determine if vitamin D supplementation is of value for prevention or as adjunctive treatment in HIV-infected persons against active tuberculosis. Dissecting the molecular mechanisms by which HIV and *M. tuberculosis* utilize autophagy has the potential to lead to the identification of novel drug candidates that can be used to prevent and treat HIV infection and related opportunistic infections including tuberculosis.

## Materials and Methods

### Ethics statement

Venous blood was drawn from HIV seronegative subjects using a protocol that was reviewed and approved by the Human Research Protections Program of the University of California, San Diego (Project 08-1613) in accordance with the requirements of the Code of Federal Regulations on the Protection of Human Subjects (45 CFR 46 and 21 CFR 50 and 56). All human studies were conducted according to the principles expressed in the Declaration of Helsinki. Written informed consent was obtained from all study participants prior to their participation.

### Isolation and cultivation of human monocyte derived macrophages

Peripheral blood mononuclear cells were isolated from whole blood by density gradient centrifugation over Ficoll-Paque Plus (GE Healthcare). Cells were then incubated overnight at 37°C, 5% CO_2_ in RPMI 1640 (Gibco) supplemented with 10% (v/v) charcoal/dextran treated, heat-inactivated fetal bovine serum (FBS; Gemini Bio-Products) and 10 ng/mL macrophage colony stimulating factor (R&D Systems), after which non-adherent cells were removed by aspiration. The same lot of FBS was used throughout all experiments. Monocyte derived macrophages were obtained by incubating the adherent population in the culture medium at 37°C, 5% CO2 for a further 10 days. The recovered cells were >95% CD163^+^, as determined by flow cytometry.

### Chemicals and inhibitors

1,25D3, pepstatin A, bafilomycin A_1_ and SID 26681509 were purchased from Sigma. Bafilomycin A_1_ and SID 26681509 were used at 100 and 50 nmol/L, respectively with pretreatment for 1 h before addition of 1,25D3 or vehicle control. Cytotoxicity of the different chemicals at the concentrations used was tested by the trypan blue dye exclusion assay, and none was found to be cytotoxic (viability was >99%).

### shRNA Transduction

MISSION small hairpin (shRNA) lentiviral particles were obtained from Sigma. Lentiviral transduction of MDMs with particles for shRNAs targeting ATG5 (SHCLNV-NM_004849/TRCN0000150940), Beclin-1 (SHCLNV-NM_003766/TRCN0000033551), CAMP (SHCLNV-NM_004345/TRCN0000118645), or scrambled non-target negative control (Scr, SHC002V) was performed according to the manufacturer's protocol. Electrophoresis and immunoblotting was as previously described [28].

### HIV and *M. tuberculosis* strains and growth conditions

HIV_Ba-L_ was obtained through the AIDS Research and Reference Reagent Program, from Suzanne Gartner and Robert Gallo [52], [53]. Virus stocks were prepared and titered as previously described using the Alliance HIV p24 antigen enzyme-linked immunosorbent assay kit (ELISA; Perkin Elmer) [54]. *M. tuberculosis* H37Rv was kindly provided by Janice Kaping, University of California San Diego. Stock strains were grown for 7–10 days to reach mid-exponential growth phase in Middlebrook 7H9 broth supplemented with 1% glycerol, 0.05% polybrene 80 and 10% OADC (oleic acid, albumin, dextrose, catalase) enrichment (all Sigma) at 37°C. Mycobacterial cultures were pelleted at 3000× g for 10 min and resuspended in Middlebrook 7H9 broth. Clumped mycobacteria were dispersed using ultrasound waves (3 to 5 min, 40 kHz; NeyTech). The sample was centrifuged at 200× g for 10 min to pellet the clumped bacilli, and the upper mycobacterial suspension was used in all experiments. Cultures were plated for viable colony forming unit (cfu) counts on Middlebrook 7H10 agar with OADC enrichment (BD Diagnostic Systems). To rule out the influence of lipopolysaccharide (LPS) in the assays, the mycobacterial suspensions were tested by the Limulus amebocyte lysate assay (Lonza). The effective LPS concentration was <2 pg/mL in experiments with mycobacteria to cell ratios of 8∶1.

### HIV and M. *tuberculosis* infection assay

5×10^5^ MDM were treated for 4 h with 1,25D3 then infected with 10^5^ TCID_50_ HIV_Ba-L_ and/or 4×10^6^ *M. tuberculosis* for 4 h. After an incubation period of 4 h, noningested *M. tuberculosis* were removed by washing four times with DPBS. The majority of extracellular mycobacteria (>99%) were removed with this process as determined by auramine-rhodamine staining. Each well was repleted with 1 mL RPMI 1640 supplemented with 10% (v/v) heat-inactivated FBS with or without 1,25D3, and incubated for 30 min (time 0) and 7 d. At day 4, 500 µL cell supernatant was sampled a replaced with 500 µL fresh media ±1,25D3 added at this point. For RNAi experiments, 1×10^5^ MDM were used and volumes and infectious particles adjusted accordingly. At the end of the incubation periods cells and supernatants were harvested and stored frozen at −70°C until further assessment in the p24 ELISA or the cfu assay.

To assess the number of intracellular mycobacteria, cells were thawed and lysed with 0.5% (w/v) sodium dodecyl sulfate (Sigma). Lysates of infected cells were resuspended vigorously, transferred into screw caps and sonicated in a preheated (37°C) water bath sonicator for 5 min (40 kHz; NeyTech). Aliquots of the lysates were diluted in Middlebrook 7H9 broth and serial dilutions of each sample were plated in quadruplicate on Middlebrook 7H10 agar with OADC enrichment (BD Diagnostics) and incubated at 37°C and 5% CO_2_ for 30 days. CFU were enumerated using a stereomicroscope. The results are expressed as mean ± SE of CFU/well.

Alternatively, a dilution of each sample was placed into a Bactec MGIT 960 (BD Diagnostics). Before inoculation, Bactec MGIT 960 tubes were supplemented as described by the manufacturer (MGIT [7-ml] package insert; Becton Dickinson). The lysates were diluted 1∶40 in Middlebrook 7H9 and a 50 µL aliquot of this was further diluted 1∶10 in Middlebrook 7H9 and inoculated into the MGIT tube (500 µL added). The tubes were introduced into the Bactec MGIT 960 instrument, as recommended by the manufacturer and incubated either until they were found to be positive by the instrument (75 Growth Units) or for 6 weeks. Results are expressed as mean time to positivity.

Aliquots of the cell supernatants were taken for HIV p24 analysis by ELISA at days 0, 4 and 7. At all time points, an aliquot of unlysed, infected cells was harvested, enumerated and viability assessed using the trypan blue exclusion assay. Recovery of the cells was >90% in all experiments, with cell viability exceeding 95% at all time points and treatments.

#### Flow cytometry

Intracellular staining of endogenous saponin resistant LC3B was performed as previously described [28], [35] using rabbit anti-LC3B (D11), mouse anti-Gag-p17 (2D11) (Abcam) and chicken anti-*M. tuberculosis* antigen 85A (Sigma) followed by allophycocyanin (APC) conjugated goat anti-rabbit IgG, fluorescein isothiocyanate (FITC) conjugated goat anti-mouse IgG1 and phycoerythrin (PE) conjugated goat anti-chicken IgY (Santa Cruz Biotechnology).

### Statistical analysis

Two-tailed, Student's *t* tests, *α* = 0.05, were used to assess whether the means of two normally distributed groups differed significantly.
